# Diabetes in Peripheral Artery Disease: Prevalence, Complications, and Polypharmacy

**DOI:** 10.3390/jcm14041383

**Published:** 2025-02-19

**Authors:** Mason Baty, Ritesh Chimoriya, Sophie James, Leonard Kritharides, Samim Behdasht, Avinash Suryawanshi, Sarah J. Aitken

**Affiliations:** 1 Faculty of Medicine and Health, The University of Sydney, Camperdown, NSW 2050, Australia; 2 Concord Institute of Academic Surgery, Concord Repatriation General Hospital, Concord, NSW 2139, Australia; 3 Department of Cardiology, Concord Hospital, The University of Sydney, 1 Hospital Road, Concord, NSW 2139, Australia; 4 Department of Pharmacy, Concord Repatriation General Hospital, Concord, NSW 2139, Australia; 5 Department of Endocrinology and Metabolism, Concord Repatriation General Hospital, Concord, NSW 2139, Australia

**Keywords:** peripheral artery disease, PAD, diabetes mellitus, cardiovascular complications, polypharmacy, diabetic control, multidisciplinary care

## Abstract

**Background**: Patients with peripheral artery disease (PAD) and diabetes face high risks of comorbidities, tissue loss, and cardiovascular events. As global type 2 diabetes (T2DM) prevalence rises, so does its incidence in symptomatic patients with PAD, though this population is under-studied in Australia. This cross-sectional analysis sought to characterize PAD patients with diabetes regarding prevalence, major complications, medication use, and prescribing patterns, comparing them to non-diabetic PAD patients. We also examined PAD complications in relation to diabetic control. **Methods**: This cross-sectional study looked at the baseline data from 105 PAD participants in the TEAM-PAD randomized controlled trial that were analyzed using descriptive statistics, prevalence odds ratios and regression analysis. Participants were recruited between June 2023 and August 2024 from public clinics, private surgeons, and Concord Repatriation General Hospital, Sydney. **Results**: Diabetes prevalence was 52.83% (n = 56) with 29.5% (n = 31) of participants with T2DM having uncontrolled hyperglycemia (HbA1c ≥ 7%), which was weakly negatively correlated with age (r = −0.372, *p* = 0.039). Participants with T2DM were twice as likely to have a history of coronary artery disease (POR 2.43; 95% with a 95% confidence interval (CI) between 1.09–5.43, and over three times as likely to have tissue loss (POR 3.39; 95% CI 1.22–9.43). The odds of polypharmacy (≥5 medications) were 10 times greater in participants with T2DM (POR 10.8; 95% CI 2.31–50.4), affecting 96.4% of this group. **Conclusions**: Diabetes prevalence and associated complications were higher than previous estimates, underscoring the challenges in managing diabetes and polypharmacy in participants with PAD. A multidisciplinary approach may improve outcomes.

## 1. Introduction

Peripheral artery disease (PAD) is a prevalent condition caused by atherosclerosis affecting lower limb arteries and is frequently associated with type 2 diabetes mellitus (T2DM) [1,2]. People with both PAD and T2DM have an increased risk of cardiovascular mortality and major amputation, as T2DM accelerates PAD progression and worsens its severity [3]. Estimates suggest that approximately one in ten patients in primary care settings have PAD [4], with the prevalence rising to one in five in patients over 75 years old [5], and of those with PAD, up to 30% may have T2DM [6,7]. These findings highlight the substantial overlap of both these conditions, leading to a heightened clinical risk in populations with PAD.

In particular, patients with both PAD and T2DM are at greater risk of cardiovascular mortality and major limb amputation compared to patients without T2DM; however, evidence is lacking regarding the amplitude of this increased risk [8,9]. There is also a substantially increased risk of mortality rates, myocardial infarction, and vascular death rates [5]. Five-year mortality rates for patients with both PAD and T2DM reach 23%, more than doubling the risk compared to patients with PAD alone, demonstrating how the presence of T2DM significantly complicates the course and outcomes of PAD [10]. Similarly, T2DM has been shown to worsen intermittent claudication by over threefold in men and over eightfold in women [7]. Patients with both diseases suffer a significantly increased risk of amputation than for either disease in isolation [8]. Thus, polypharmacy is highly prevalent due to the need to manage multiple conditions, including glycaemic control, cardiovascular risk, and PAD symptoms [11,12,13]. Although necessary for addressing these complex needs, polypharmacy also elevates the risk of adverse drug interactions, medication non-adherence, and overall treatment burden, complicating effective disease management [14,15].

Despite the rising global prevalence of PAD and T2DM, there is very limited research focused on the unique risks, treatment needs, and complications in this overlapping patient group. Patients with both PAD and T2DM experience significantly poorer outcomes, yet gaps remain in understanding their specific risk factors, glycaemic control, and pharmacological management. Our study aims to describe and compare the baseline clinical and demographic characteristics of patients with PAD and T2DM, identify the associations between PAD and T2DM, and analyze the prescribing patterns.

## 2. Materials and Methods

This cross-sectional study was conducted at a tertiary care center in Sydney, NSW. This was a cross-sectional analysis of baseline data collected from the ongoing TEAM-PAD randomized controlled trial (RCT) between June 2023 and August 2024. The detailed sampling methodology and procedure are described elsewhere in the original trial protocol [16]. The details on the RCT are available on the Australia New Zealand Clinical Trial Registry (Registration number: ACTRN12623000995673) [17]. This study recruited participants with PAD from publicly funded outpatient clinics, private consulting rooms of affiliate surgeons, or inpatient settings. The larger, ongoing RCT evaluated the efficacy of a multidisciplinary team-based intervention on cardiovascular risk in participants with PAD. The TEAM-PAD trial aims to demonstrate a difference in composite cardiovascular risk at the conclusion. This current study only includes participants who completed the baseline clinical assessments during the study period. This study was prepared and presented in accordance with the STROBE checklist for observational cross-sectional studies, which has been included in Supplementary Table S1 [18]. This study was approved by the Sydney Local Health District—Concord Hospital HREC, protocol number 2023/ETH00929, on 28 July 2023. This study was conducted in accordance with the Declaration of Helsinki.

### 2.1. Study Population

The participants included in this study were adults (≥18 years of age) with a diagnosis of PAD, which was defined as having an Ankle Brachial Index (ABI) of less than 0.9, a Toe Brachial Index (TBI) of less than 0.6, a history of vascular surgical or endovascular intervention for PAD, or a confirmed diagnosis via medical imaging or clinical grading. All participants were receiving care for PAD at the site of this study and provided consent for study participation. Those who were unable to provide consent due to a lack of capacity or who declined to participate were excluded. Additionally, those with an estimated life expectancy of less than one year or a diagnosis of terminal illness were also excluded from participation. No participants in this study had a diagnosis of type 1 diabetes mellitus.

### 2.2. Data Collection and Variables

Participant data were collected prospectively by trained clinical researchers according to a predefined proforma. These data included a comprehensive clinical examination, which included measuring blood pressure, an anthropometric assessment, palpation of the peripheral pulses, and examination for ulceration and gangrene. Additionally, detailed verbal patient-reported medical history was obtained through a standardized health questionnaire covering demographics, medical history, medications, allergies, family, and social history, and the collected data were verified with electronic medical records (eMR) that monitored hospital admissions, surgical procedures, and any adverse events. Routine radiological, pathological, and biochemical investigations included vascular assessments (electrocardiograph and ABI), blood tests (full blood count, electrolytes, urea, creatinine, fasting lipids, HbA1c, kidney function test, and liver function tests), and urine tests (protein, microalbumin, and creatinine). The participants were stratified into those with T2DM and those without a diagnosis of T2DM. Glycaemic control was defined as ‘controlled’ if the Hemoglobin A1c (HbA1c) was less than 7% or ‘uncontrolled’ if HbA1c was 7% or higher. Similarly, tissue loss was defined as current or past ulceration, gangrene, necrosis, or a history of amputation. Polypharmacy and hyper-polypharmacy were defined as five or more medications and ten or more regular medications per day, respectively. The covariates included in this analysis were chosen because of their known clinical associations with complications of PAD and diabetes. Pre-existing clinical diagnoses (hypertension, dyslipidemia, coronary artery disease, cerebrovascular disease, heart failure, and atrial fibrillation) were diagnoses reported by the patient, confirmed with their eMR record. Clinical examination parameters (systolic BP) and laboratory tests were collected at the time of study enrolment.

### 2.3. Primary and Secondary Outcomes

The primary outcome of interest was the variation in glycemic control observed in participants with T2DM and PAD, as measured by the HbA1c. The participants were classified as having ‘uncontrolled’ hyperglycemia if their baseline HbA1c was >7. Secondary outcomes included factors influencing the differences in cardiovascular risk and outcomes between participants with T2DM and those without, the number of pharmacological agents prescribed to participants with T2DM, and the burden of polypharmacy. Polypharmacy was measured by the total number of regular medications, with polypharmacy defined as 5 or more regular medications and hyper-polypharmacy defined as 10 or more medications.

### 2.4. Statistical Analysis

The R software, version 4.4.0 (R Foundation for Statistical Computing, Vienna, Austria), was used for the statistical analysis. Descriptive statistics of the participant characteristics were calculated, and medians and percentages were presented for all relevant variables. Frequencies of the patient characteristics were tabulated, and the prevalence was determined by calculating the percentages for each category. A Shapiro–Wilk test was performed to assess the normality of the distribution of the continuous data. Normally distributed continuous data were compared using the independent *t*-test, whereas non-normally distributed data were compared using the Mann–Whitney U test. Categorical variables were compared using the chi-squared test to determine the differences between groups. Logistic regression analysis was utilized to calculate the prevalence odds ratios and 95% confidence intervals (CI) to compare participant groups, which were adjusted for age and gender. All tests with *p*-values < 0.05 were considered statistically significant.

## 3. Results

This study included 105 participants with PAD who had completed baseline assessments at the time of analysis. As shown in Table 1, the median age of the participants was 72 years old, with 33.3% (n = 35) of the cohort being women. Altogether, 52.83% (n = 56) of the participants had T2DM. The participant characteristics and comorbidities at the time of enrolment in the TEAM-PAD study have been summarized in Table 1.

### 3.1. Characteristics of Participants with T2DM Compared to Those Without Diabetes

As shown in Table 1, there were no differences between the groups in terms of age and sex (*p* = 0.075 and *p* = 0.628, respectively). In terms of comorbidities, the participants with T2DM had a higher prevalence of a history of hypertension (91.1% vs. 69.4%; *p* = 0.011). Additionally, tissue loss was substantially more common among individuals with T2DM (34% vs. 8.1%; *p* = 0.028) than those with PAD alone. The participants without T2DM had a higher HDL and LDL (1.2 mmol/L vs. 1.1 mmol/L; *p* = 0.008 and 2.3 mmol/L vs. 1.8 mmol/L; *p* = 0.021, respectively). The proportion of participants who were currently smoking was higher among those without T2DM compared to those with T2DM (34.7% vs. 16.1%; *p* = 0.040).

### 3.2. Association with Poor Glycaemic Control in Participants with T2DM

**Figure 1:**
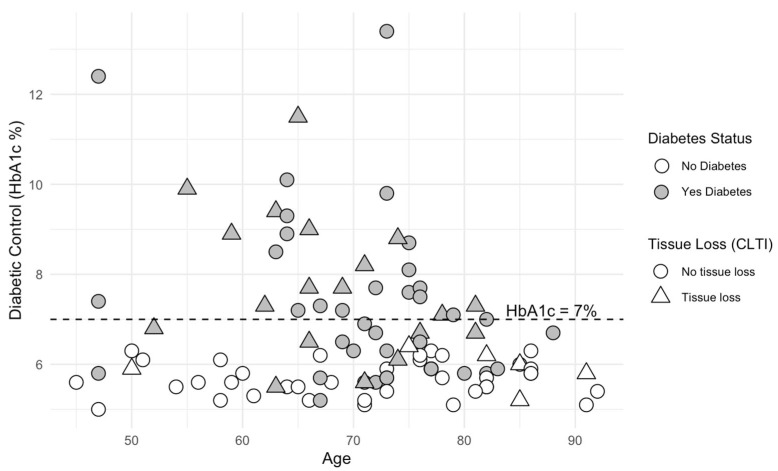
Scatterplot comparing diabetic control as measured by baseline HbA1c against the age of participants. The history of tissue loss is indicated by triangular symbols. Participants with uncontrolled T2DM are placed above the dotted line equivalent to an HbA1c of 7% or greater.

In terms of glycemic control, 55.3% (n = 31) of the participants with T2DM had a HbA1c ≥ 7% at the time of the study assessment and were classified as having ‘uncontrolled’ hyperglycemia. Figure 1 demonstrates the distribution of HbA1c in all participants. The participants with uncontrolled HbA1c tended to be younger. There was a weak negative correlation between age and the HbA1c (%) (r = −0.372, *p* = 0.0394).

Table 2 shows that the prevalence of tissue loss (including ulcers, gangrene, and amputations) is higher in participants with T2DM compared to those without. Tissue loss affected 32.1% (n = 18) of the participants with T2DM compared to 12.2% (n = 6) of the participants without T2DM (*p* = 0.028). Wet gangrene affected 16.1% (n = 9) of the participants with T2DM compared to 2% (n = 1) of those without T2DM (*p* = 0.034).

**Figure 2:**
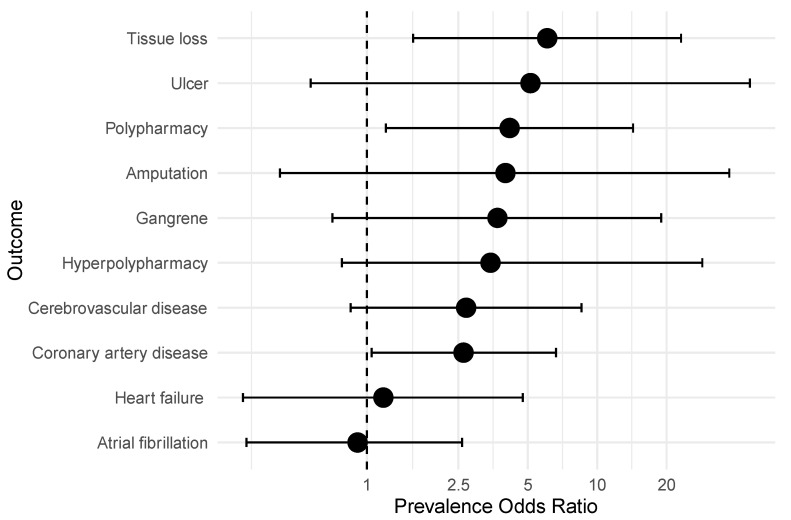
Forest plot of prevalence odds ratios of polypharmacy and tissue loss in participants with T2DM compared to those without T2DM. The x-axis uses a logarithmic scale. Adjusted using age and gender.

Figure 2 demonstrates a forest plot of the prevalence odds ratios showing the prevalence of tissue loss across the group. The participants with T2DM were three times more likely to have had any type of tissue loss compared to the participants without T2DM (POR 3.6; 95% CI 1.28–10). The participants with T2DM were 12 times more likely to be taking five or more medications compared to those without T2DM (POR 12.82; 95% CI 2.92–55.6).

### 3.3. Medications for Diabetes and Polypharmacy

Table 3 describes the medications prescribed to all participants. Acetylsalicylic acid (n = 71, 67.6%) and statins (n = 72, 68.5%) were the most common medications prescribed in the total patient group and were equally prevalent in participants with and without T2DM. The participants with T2DM were more likely to be prescribed two agents for dyslipidemia than those without T2DM.

The participants with T2DM were taking more medications overall than the participants with only PAD. The median number of medications was 11 (IQR 5.2) in the group with T2DM compared to 6 (IQR 5) in the group without T2DM (*p* ≤ 0.001). Polypharmacy was observed in 96.4% (n = 54) of the participants with T2DM compared to 71.4% (n = 35) in the participants without T2DM (*p* ≤ 0.01). Hyper-polypharmacy (≥10 regular medications) was reported in 64.2% (n = 32) of the participants with T2DM compared to 24.4% (n = 12) in the participants without T2DM. The participants with T2DM had 12 times greater odds of taking five or more medications compared to those without T2DM (POR 12.82; 95% CI 2.92–55.6).

In terms of antiglycemic medications prescribed for participants with T2DM, metformin was the most common drug prescribed (n = 31, 55.3%), followed by insulin (n = 24, 42.8%), and then SGLT2 inhibitors (n = 20, 35.7%). GLP1 agonists were prescribed to 23.2% (n = 13) of the participants while DPP4 inhibitors were prescribed to 21.4% (n = 12).

## 4. Discussion

Our findings highlight significant differences between patients with PAD who have T2DM and those without T2DM. Patients with T2DM are more likely to have a history of tissue loss and cardiovascular complications and have higher rates of polypharmacy compared to patients without T2DM. This increases the baseline cardiovascular risk profile for patients with both T2DM and PAD and exposes them to future adverse health events. Whilst dyslipidemia medications and antiglycemics are frequently prescribed, many patients with T2DM are missing out on best-practice antithrombotic medications [19]. HbA1c was poorly controlled in over half the cohort with T2DM, exposing them to future risks, and is linked to higher associations with tissue loss.

Cardiovascular disease is more likely to be present in patients with PAD and T2DM compared to patients without T2DM, as shown in our study. This finding is consistent with previous studies, which show that T2DM within the context of PAD confers two to three times the risk of cardiovascular disease [20,21]. The many macrovascular effects of chronic diabetes are well understood, and this is reflected in this population of patients as well as other risk factors. The elevated risks associated with the combination of PAD and coronary artery disease require additional attention in patients with T2DM. For example, in our cohort, patients with T2DM had a prevalence of hypertension greater than those without T2DM. Yet, the observed relative risk of hypertension of approximately 1.5 was lower than is seen when patients with T2DM are compared to the general population when the risk is closer to double [22]. This is likely due to the high baseline prevalence of hypertension in patients with PAD. The very high prevalence of hypertension seen in the cohort with T2DM reinforces that many patients with PAD are at a very high risk of major cardiovascular events and disease progression due to uncontrolled risk factors [23].

Current smoking was more than twice as prevalent in participants without T2DM than those with T2DM, while the prevalence of past smoking was very similar between these groups. This difference suggests that participants with a diagnosis of T2DM were more likely to have given up smoking than those without T2DM [24]. This is a positive sign for smoking management in patients with T2DM but also highlights the need for similar improvements in patients without T2DM, as smoking remains a significant risk factor for PAD. This is equally important for other lifestyle choices, such as dietary habits, physical activity, and alcohol consumption, which may compound the effects of uncontrolled T2DM and progressing PAD [25,26,27].

The prevalence of uncontrolled diabetes within our cohort is consistent with the rates seen in recent studies looking at glycemic control in other cohorts with diabetes, where the prevalence of uncontrolled diabetes is up to 50% [28]. Many of the same problems affecting diabetic control in patients with PAD are like those of other cohorts with T2DM, such as health education and awareness, access to healthcare, and medication adherence [29,30,31]. The high prevalence of T2DM and uncontrolled T2DM participants with PAD underscores significant implications for disease progression and complications, as our findings revealed that patients with T2DM were at least three times more likely to experience greater and more severe tissue loss compared to those without T2DM. Australian data for this outcome are limited; however, one American study has shown that rates of ischemic ulcers and gangrene are more commonly seen in patients with overlapping diabetes and PAD, and a history of amputation is around five times as likely [32]. Concomitant PAD and T2DM have a negative synergistic effect on PAD progression and severity by worsening neuropathy and infection with reduced wound healing from decreased blood supply, increasing the likelihood of tissue loss [33]. Previous studies have shown that poor glucose control increased severe manifestations of PAD, including the need for revascularization and symptoms of intermittent claudication, by up to five times [34]. Other studies demonstrate that glycemic control in patients with T2DM was more strongly associated with microvascular disease in patients with PAD [35]. The increased risk of multisite atherosclerotic disease observed in this study and others reinforces the need for tight glycemic control to reduce the risk of severe tissue loss-related complications in patients with both PAD and T2DM.

As demonstrated in our study, participants with PAD and T2DM used more medications on average, with over 95% experiencing polypharmacy (taking five or more regular medications) and over half experiencing hyper-polypharmacy (taking 10 or more medications), compared to those with PAD alone. Previous studies show polypharmacy in up to 78% of diabetes patients, increasing with age, with most estimates around 30–60% [36,37]. The higher rates seen in this study can be explained due to the background of PAD and older patient cohorts. Patients with T2DM had more than 12 times greater odds of polypharmacy. Studies have shown that polypharmacy is linked to a range of negative clinical outcomes, including drug–drug interactions, medication non-adherence, adverse drug events, falls, functional decline, and mortality [13,38,39,40,41]. There was a lower rate of prescribing guideline-advocated antithrombotic medications for patients with T2DM compared to those without T2DM. This emphasizes the importance of balancing the best medical therapy against the risks of polypharmacy in these patients. On the one hand, patients with poor glycemic control have been shown to have an increased risk of developing and worsening the symptoms of PAD, such as claudication, with every 1% increase in HbA1c as well as an increased risk of PAD-related hospitalizations [32,34]. But, other studies have also suggested that targeting an HbA1c between 7.5% and 9% will maximize benefits and minimize the harms of polypharmacy in elderly patients with diabetes [42]. The management of these complex patients should be careful and individualized and would be well suited to a multidisciplinary approach.

This study addresses an important knowledge gap in the real-world management of patients with the combined cardiovascular risks of T2DM and PAD. This group is poorly investigated, especially in Australia. This cross-sectional analysis has identified important areas of future research; however there are some limitations inherent to the study design and demographics and geographical location where the study was conducted. The prevalence of T2DM was almost ten times higher in this cohort of patients with PAD than in current estimates of the Australian general population [43,44]. Overseas studies report higher prevalence rates between 20–30 percent [6,45], which were closer to what was observed but still significantly less than this cohort. This higher recruitment of patients with T2DM may lead to a selection bias, as we primarily recruited patients from public hospital clinics, which prioritize patients with more severe PAD and have a high cross-over with high-risk diabetes foot clinics. The higher prevalence seen in this study could also be due to the increasing diabetes epidemic seen globally and in Australia [46]. The prevalence of T2DM has also been increasing in patients with symptomatic PAD worldwide [1,47]. It is possible that the findings of this study are more closely aligned with a prevalence of T2DM seen in patients with PAD in Australia than those previously suggested. However, it is likely to still be an overestimate of the exact prevalence due to the large portion of asymptomatic patients with PAD, which has been shown to be of high prevalence in Australian healthcare settings [48]. The participants recruited for this study were recruited from areas within the Sydney metropolitan area, so it is unlikely to be representative of rural or remote communities in Australia. The study population had twice as many men as women. Our study underscores the need for a larger, well-powered study to better understand the prevalence of T2DM, polypharmacy, and related complications within the Australian PAD population, ideally involving collaboration across hospitals, outpatient clinics, and general practices. Future studies should also consider examining the clinical severity of PAD alongside the detailed anatomical distribution of atherosclerotic lesions between subgroups with and without T2DM to provide a more comprehensive understanding of the disease pathology and its impact on patient outcomes.

## 5. Conclusions

Our study reveals a higher-than-expected prevalence of T2DM and associated complications, including increased rates of hypertension, tissue loss, and elevated polypharmacy compared to previous Australian estimates. These findings emphasize the critical need for effective management strategies for T2DM to mitigate the risks of major cardiovascular events and tissue loss. The high prevalence of polypharmacy within this patient group further underscores the complexities of their care, presenting a significant challenge for vascular surgeons and general practitioners. These findings suggest that optimizing care for this vulnerable population may benefit from multidisciplinary approaches, potentially improving outcomes through collaborative management and tailored interventions.

## Figures and Tables

**Table 1:** Baseline characteristics of study participants.

Variable Median (IQR or n (%))	All Participants (n = 105)	Without T2DM (n = 49)	with T2DM (n = 56)	*p*-Value
Sociodemographic characteristics				
Age (years)	72 (IQR 14)	76 (IQR 18)	71 (IQR 10.5)	0.075
Female	35 (33.3%)	18 (36.7%)	17 (30.3%)	0.628
Anthropometry, behavioral factors, and pre-existing comorbidities				
BMI (kg/m^2^)	27.5 (IQR 5.2)	27.9 (IQR 5.4)	26.5 (IQR 5.5%)	0.228
Hypertension	85 (80.9%)	34 (69.4%)	51 (91.1%)	0.011
Dyslipidemia	83 (79%)	37 (75.5%)	46 (82.1%)	0.500
Uncontrolled hyperglycemia	31 (29.5%)	0 (0%)	31 (55.3%)	-
Coronary artery disease	44 (41.9%)	15 (30.6%)	29 (51.7%)	0.045
Cerebrovascular disease	24 (22.8%)	7 (14.2%)	17 (30.3%)	0.083
Heart failure	12 (11.4%)	4 (8.2%)	8 (14.2%)	0.490
Atrial fibrillation	21 (20%)	9 (18.3%)	12 (21.4%)	0.880
Current smoker	26 (24.7%)	17 (34.7%)	9 (16.1%)	0.040
Previous smoker	50 (47.6%)	22 (44.9%)	28 (50%)	0.740
Never smoked	28 (26.6%)	10 (20.4%)	28 (32.1%)	0.250
Clinical examination and biochemical profile				
HbA1c (%)	6.1 (IQR 1.7)	5.7 (IQR 0.6)	7.2 (IQR 2.4)	<0.001
Systolic blood pressure (mmHg)	138.6 (IQR 18)	135 (IQR 15.5)	138 (IQR 18.4)	0.287
eGFR (mL/min/1.73 m^2^)	70.5 (IQR 33.7)	70.5 (IQR 30.5)	71 (IQR 36.2)	0.890
HDL (mmol/L)	1.1 (IQR 0.6)	1.2 (IQR 0.6)	1.1 (IQR 0.4)	0.008
LDL (mmol/L)	1.8 (IQR 1.1)	2.3 (IQR 1.4)	1.8 (IQR 0.8)	0.021
Triglycerides (mmol/L)	1.4 (IQR 1.2)	1.4 (IQR 1.2)	1.4 (IQR 1.1)	0.510
ALP (IU/L)	78 (IQR 43)	77.5 (IQR 22.5)	85 (IQR 46)	0.785
GGT (IU/L)	25 (IQR 28)	24 (IQR 17.7)	30 (IQR 25.48)	0.741
ALT (IU/L)	22 (IQR 10)	24.5 (IQR 8.5)	16 (IQR 11)	0.540
AST (IU/L)	22 (IQR 10)	24.5 (IQR 8.5)	20 (IQR 8)	0.021

Abbreviation: ALT: alanine aminotransferase; AST: aspartate aminotransferase; BMI: body mass index; GGT: gamma-glutamyl transferase; eGFR: estimated glomerular filtration rate; HDL: high-density lipoprotein; LDL: low-density lipoprotein; T2DM: type 2 diabetes mellitus.

**Table 2:** Prevalence of tissue loss (either ulcer, gangrene, amputation) in the study population. Values in percentage values. *P*-values were calculated comparing T2DM and no T2DM using chi^2^ analysis.

Variable Median n (%)	All Participants (n = 105)	Without T2DM (n = 49)	With T2DM (n = 56)	*p*-Value
Tissue loss	24 (22.8%)	6 (12.2%)	18 (32.1%)	0.028
Ulcer	6 (5.7%)	1 (2%)	5 (8.9%)	0.273
Dry gangrene	2 (1.9%)	0 (0%)	2 (3.6%)	-
Wet gangrene	10 (9.52%)	1 (2%)	9 (16.1%)	0.034
Amputation	5 (4.76%)	1 (2%)	4 (7.1%)	0.440

**Table 3:** Prescribed medications in the study participants. *P*-values were calculated using chi^2^ analysis. *p*-values for non-parametric continuous data were calculated using the Mann–Whitney U test.

Variable Median (IQR or n (%))	Total Participants (n = 105)	Without T2DM (n = 49)	with T2DM (n = 56)	*p*-Value
Total number of medications	9 (IQR 7)	6 (IQR 5)	11 (IQR 5.2)	<0.001
Polypharmacy	89 (84.7%)	35 (71.4%)	54 (96.4%)	0.001
Hyper-polypharmacy	48 (45.7%)	12 (24.4%)	36 (64.2%)	<0.001
Lipid-lowering medication				
Rosuvastatin	41 (39%)	18 (36.7)	23 (41.1%)	0.799
Atorvastatin	31 (29.5%)	15 (30.6)	16 (28.5%)	0.988
Simvastatin	7 (6.6%)	3 (6.1%)	4 (7.2%)	
Pravastatin	1 (0.9%)	0 (0)	1 (1.7%)	-
Fenofibrate	12 (11.4%)	2 (4.1)	10 (17.8%)	0.056
Ezetimibe	10 (9.5%)	5 (10.2)	5 (8.9%)	1
PCSK9 inhibitor	1 (0.9%)	0 (0)	1 (1.7%)	-
Antithrombotic medications				
Acetylsalicylic acid	71 (67.6%)	32 (65.3%)	39 (69.6%)	0.791
Clopidogrel	17 (16.2%)	9 (18.3%)	8 (14.2%)	0.763
Apixaban	18 (17.1%)	10 (20.4%)	8 (14.2%)	0.568
Dabigatran	0 (0%)	0 (0%)	0 (0%)	-
Rivaroxaban low-dose	17 (16.2%)	11 (22.4%)	6 (10.7%)	0.179
Rivaroxaban full-dose	7 (6.6%)	3 (6.1%)	4 (7.1%)	1
Antiglycemic medication				
Metformin	31 (29.5%)	0 (0%)	31 (55.3%)	-
Sulfonylureas	7 (6.6%)	0 (0%)	7 (12.5%)	-
SGLT2 inhibitor	20 (19%)	0 (0%)	20 (35.7%)	-
GLP1 agonist	13 (12.3%)	0 (0%)	13 (23.2%)	-
DPP4 inhibitor	12 (11.4%)	0 (0%)	12 (21.4%)	-
Insulin	24 (22.8%)	0 (0%)	24 (42.8%)	-

Abbreviation: DPP4: dipeptidyl peptidase-4 inhibitor; GLP1: glucagon-like peptide 1 agonist; Insulin: a type of antiglycemic medication; IQR: interquartile range; LDL: low-density lipoprotein; PCSK9: proprotein convertase subtilisin/kexin type 9 inhibitor; SGLT2: sodium-glucose co-transporter 2 inhibitor; T2DM: type 2 diabetes mellitus.

## Data Availability

The data presented in this study are available on request from the corresponding author and are subject to approval. The data are not publicly available due to privacy.

## Supplementary Materials

The following supporting information can be downloaded at: https://www.mdpi.com/article/10.3390/jcm14041383/s1, Table S1: STROBE Statement—Checklist of items that should be included in reports of cross-sectional studies.
