# Supplementary material for: Diabetes in Peripheral Artery Disease: Prevalence, Complications, and Polypharmacy
Source: J Clin Med. 2025 Feb 19;14(4):1383. doi: 10.3390/jcm14041383 (PMC11856835; doi:10.3390/jcm14041383)
Supplement: Supplementary file 1 [file jcm-14-01383-s001.zip › jcm-3414208-supplementary.pdf]

Diabetes in Peripheral Artery Disease: Prevalence, Complications, and Polypharmacy

Mason Baty [1], Ritesh Chimoriya [1,2,*], Sophie James [1,2], Leonard Kritharides [1,3], Samim Behdasht [4], Avinash Suryawanshi [5] and Sarah J. Aitken [1,2,*] on behalf of TEAM-PAD Collaborators [†]

[1] Faculty of Medicine and Health, The University of Sydney, Camperdown, NSW 2050, Australia
[2] Concord Institute of Academic Surgery, Concord Repatriation General Hospital, Concord, NSW 2139, Australia
[3] Department of Cardiology, Concord Hospital, The University of Sydney, 1 Hospital Road, Concord, NSW 2139, Australia
[4] Department of Pharmacy, Concord Repatriation General Hospital, Concord, NSW 2139, Australia
[5] Department of Endocrinology and Metabolism, Concord Repatriation General Hospital, Concord, NSW 2139, Australia
* Correspondence: ritesh.chimoriya@health.nsw.gov.au (R.C.); sarah.aitken@sydney.edu.au (S.J.A.)
† TEAM-PAD collaborators are provided in the Supplementary Materials.

Diabetes in Peripheral Artery Disease: Prevalence, Complications and Polypharmacy

TEAM-PAD collaborators: Associate Professor Sarah Aitken (Principal Investigator), Professor Leonard Kritharides, Dr. Sophie James, Dr. Ritesh Chimoriya, Ms. Jasmine Chan, Dr. Hamid Hajian, Professor Steven Twigg, Dr. Janani Thillainadesan, Dr. Saundeep Sen, Dr. Avinash Suryawanshi, Professor Vasikaran Naganathan, Mr. Samim Behdasht, Dr. Hue Davis, Dr. Amy Kitajima, Dr. Marco LeeSolano, Dr. Hayden Ly, Dr. Jacky Loa, Associate Professor Michelle Cunich, Dr. Richard Kerdic, Dr. Robert Tang, Dr. Saissan Rajendran, Associate Professor Mary Kavurma, Dr. Nima Iranpour, Ms. Naomi Cooper, Ms. Wen Xin Chan and Mr Mason Baty.

Supplementary table S1: STROBE Statement—Checklist of items that should be included in reports of ***cross-sectional studies***

	Item No	Recommendation	Location in Manuscript
**Title and abstract**	1	(*a*) Indicate the study's design with a commonly used term in the title or the abstract	Abstract
		(*b*) Provide in the abstract an informative and balanced summary of what was done and what was found	Abstract
**Introduction**			
Background/rationale	2	Explain the scientific background and rationale for the investigation being reported	Section 1
Objectives	3	State specific objectives, including any prespecified hypotheses	Section 1
**Methods**			
Study design	4	Present key elements of study design early in the paper	Section 2
Setting	5	Describe the setting, locations, and relevant dates, including periods of recruitment, exposure, follow-up, and data collection	Section 2, 2.1, 2.2 and 2.3
Participants	6	(*a*) Give the eligibility criteria, and the sources and methods of selection of participants	Section 2.1
Variables	7	Clearly define all outcomes, exposures, predictors, potential confounders, and effect modifiers. Give diagnostic criteria, if applicable	Section 2.2 and 2.3
Data sources/	8*	For each variable of interest, give sources of data and details of	Section 2.2

measurement		methods of assessment (measurement). Describe comparability of assessment methods if there is more than one group	and 2.3
Bias	9	Describe any efforts to address potential sources of bias	Section 2.4
Study size	10	Explain how the study size was arrived at	Section 2
Quantitative variables	11	Explain how quantitative variables were handled in the analyses. If applicable, describe which groupings were chosen and why	Section 2.4
Statistical methods	12	(*a*) Describe all statistical methods, including those used to control for confounding	Section 2.4
		(*b*) Describe any methods used to examine subgroups and interactions	Section 2.4
		(*c*) Explain how missing data were addressed	N/A
		(*d*) If applicable, describe analytical methods taking account of sampling strategy	N/A
		(*e*) Describe any sensitivity analyses	N/A
**Results**			
Participants	13*	(a) Report numbers of individuals at each stage of study—eg numbers potentially eligible, examined for eligibility, confirmed eligible, included in the study, completing follow-up, and analysed	Section 3
		(b) Give reasons for non-participation at each stage	N/A
		(c) Consider use of a flow diagram	N/A
Descriptive data	14*	(a) Give characteristics of study participants (eg demographic, clinical, social) and information on exposures and potential confounders	Section 3.1
		(b) Indicate number of participants with missing data for each variable of interest	
Outcome data	15*	Report numbers of outcome events or summary measures	Section 3.1 and 3.2
Main results	16	(*a*) Give unadjusted estimates and, if applicable, confounder-adjusted estimates and their precision (eg, 95% confidence interval). Make clear which confounders were adjusted for and why they were included	Section 3.1 and 3.2
		(*b*) Report category boundaries when continuous variables were categorized	N/A
		(*c*) If relevant, consider translating estimates of relative risk into absolute risk for a meaningful time period	N/A
Other analyses	17	Report other analyses done—eg analyses of subgroups and interactions, and sensitivity analyses	Section 3.1 and 3.2
**Discussion**			
Key results	18	Summarise key results with reference to study objectives	Section 4
Limitations	19	Discuss limitations of the study, taking into account sources of potential bias or imprecision. Discuss both direction and magnitude of any potential bias	Section 4
Interpretation	20	Give a cautious overall interpretation of results considering objectives, limitations, multiplicity of analyses, results from similar studies, and other relevant evidence	Section 4
Generalisability	21	Discuss the generalisability (external validity) of the study results	Section 4
**Other information**			

Funding	22	Give the source of funding and the role of the funders for the present study and, if applicable, for the original study on which the present article is based	Funding

*Give information separately for exposed and unexposed groups.

Note: An Explanation and Elaboration article discusses each checklist item and gives methodological background and published examples of transparent reporting. The STROBE checklist is best used in conjunction with this article (freely available on the Web sites of PLoS Medicine at http://www.plosmedicine.org/, Annals of Internal Medicine at http://www.annals.org/, and Epidemiology at http://www.epidem.com/). Information on the STROBE Initiative is available at www.strobe-statement.org.
